# Distinct TDP-43 inclusion morphologies in frontotemporal lobar degeneration with and without amyotrophic lateral sclerosis

**DOI:** 10.1186/s40478-017-0480-2

**Published:** 2017-10-27

**Authors:** Rachel H. Tan, Yue Yang, Woojin S. Kim, Carol Dobson-Stone, John B. Kwok, Matthew C. Kiernan, Glenda M. Halliday

**Affiliations:** 1 Brain and Mind Centre, Sydney Medical School, The University of Sydney, 94 Mallett Street, Camperdown, NSW 2050 Australia; 2 School of Medical Sciences, University of New South Wales, & Neuroscience Research Australia, Sydney, Australia; 3 Department of Neurology, Royal Prince Alfred Hospital, Sydney, Australia

**Keywords:** TDP-43 pathology, Neuronal cytoplasmic inclusions, Morphology, Frontotemporal lobar degeneration, Amyotrophic lateral sclerosis

## Abstract

The identification of the TAR DNA-binding protein 43 (TDP-43) as the ubiquitinated cytoplasmic inclusions in frontotemporal lobar degeneration (FTLD) and amyotrophic lateral sclerosis (ALS) confirmed that these two diseases share similar mechanisms, likely to be linked to the abnormal hyperphosphorylation, ubiquitination and cleavage of pathological TDP-43. Importantly however, a quantitative analysis of TDP-43 inclusions in predilection cortical regions of FTLD, FTLD-ALS and ALS cases has not been undertaken. The present study set out to assess this and demonstrates that distinct TDP-43 inclusion morphologies exist in the anterior cingulate cortex, but not the motor cortex of FTLD and FTLD-ALS. Specifically, in the anterior cingulate cortex of FTLD cases, significant rounded TDP-43 inclusions and rare circumferential TDP-43 inclusions were identified. In contrast, FTLD-ALS cases revealed significant circumferential TDP-43 inclusions and rare rounded TDP-43 inclusions in the anterior cingulate cortex. Distinct TDP-43 inclusion morphologies in the anterior cingulate cortex of FTLD and FTLD-ALS may be linked to heterogeneity in the ubiquitination of pathological TDP-43 inclusions, with the present study providing evidence to suggest the involvement of distinct pathomechanisms in these two overlapping clinical syndromes.

## Introduction

Neuronal cytoplasmic aggregates containing the nuclear TAR DNA-binding protein 43 (TDP-43) are characteristically observed in the cortical neurons of frontotemporal lobar degeneration (FTLD) with TDP-43 pathology (FTLD-TDP), and in the lower motor neurons of cases with amyotrophic lateral sclerosis (ALS). The presence of this shared pathological protein has reinforced the theory that FTLD and ALS represent two ends of a disease continuum, with recent studies attributing the manifestation of this pathological protein in two distinct clinical syndromes to its accumulation in key brain regions [1, 8, 12, 13, 25, 30]. Based on the morphology and distribution of cortical TDP-43 pathology, FTLD cases are routinely classified into one of four pathological subtypes (FTLD-TDP Type A-D) [21, 27], with Types A and B the most commonly identified in the ~20% of FTLD cases with ALS (FTLD-ALS) [8, 17, 22, 27]. Although TDP-43 pathology is identified in the frontal cortices of ~70% of ALS cases without FTLD [9], these cases are not routinely subtyped according to the FTLD classification scheme, with a novel TDP-43 classification scheme recently proposed for ALS [29]. Given the wide acceptance of the FTLD-ALS continuum theory, it is not clear why cortical TDP-43 pathology would be classified according to two separate schemes, particularly since cortical TDP-43 pathology identified in Alzheimer’s disease cases (AD) are classified as per in FTLD [4, 33]. Importantly, a quantitative analysis comparing the morphology of cortical TDP-43 pathology in FTLD, FTLD-ALS and ALS cases has not been performed. The present study set out to do this in order to determine if cortical TDP-43 inclusion morphology is similar in these three clinicopathological groups believed to represent a disease continuum.

## Materials and methods

### Case selection

All cases with a pathological diagnosis of FTLD-TDP and/or ALS-TDP were selected from a neuropathological series collected by the Sydney Brain Bank through regional brain donor programs in Sydney, Australia. The brain donor programs hold approval from the Human Research Ethics Committees of the University of New South Wales and comply with the statement on human experimentation issued by the National Health and Medical Research Council of Australia. Patients were diagnosed during life by experienced clinicians using standard clinical diagnostic criteria [2, 16, 19, 24, 26, 34] following a medical interview, cognitive testing, and informant history. Standardized neuropathological characterization was performed [10, 23] and all ALS cases demonstrated upper and lower motor neuron degeneration accompanied by TDP-43 neuronal inclusions in surviving motor neurons. Given the specific focus on pathological TDP-43 inclusions and the FTLD-ALS continuum, FTLD-TDP type C cases were excluded. A total of 61 cases met these inclusion criteria, and were comprised of 46 cases with FTLD-TDP (of which 50% (*n* = 23) also fulfilled pathological criteria for ALS) and 15 ALS cases (Table 1). All cases had been screened for mutations in the *C9ORF72* and *GRN* genes using previously published methods [11, 28] and a pathogenic mutation was identified in FTLD cases only (43% *C9ORF72* expansions (*n* = 20) and 17% *GRN* mutations (*n* = 8)). This research project was approved by the Human Research Ethics Committees of the University of New South Wales.

**Table 1:** Demographic, clinicopathological and genetic profile of cases

	FTLD	FTLD-ALS	ALS
N (% male)	23 (52%)	23 (57%)	15 (53%)
Age at death (year)	65 ± 8^a^	67 ± 8^a^	71 ± 8
Age at onset (year)	59 ± 8^a^	63 ± 8^a^	70 ± 7
Disease duration (year)	6 ± 4^a^	4 ± 3	2 ± 2
Postmortem delay (hours)	25 ± 23	25 ± 17	22 ± 10
bvFTD/SD/PNFA/FTD unspecified/AD (n)	21/0/0/1/1	17/2/2/1/1	N/A
*C9ORF72* carrier % (n)	48% (11)^a^	39% (9)^a^	0% (0)
*GRN* carrier % (n)	35% (8)^a^	0% (0)^b^	0% (0)^b^

*DD* Disease duration (years), *DO* Disease onset (years), *PM*D postmortem delay (hours), *bvFTD* behavioral variant frontotemporal dementia, *SD* semantic dementia, *PNFA* progressive non-fluent aphasia, *AD* Alzheimer’s disease, *N/A* not applicable.^a^ *p* < 0.05 compared to ALS, ^b^ *p* < 0.05 compared to FTLD

### Brain regions assessed

The selection of the anterior cingulate cortex and motor cortex as the cortical regions-of-interest in this study was based on these regions being the predilection sites and early cortical regions targeted by TDP-43 aggregates in FTLD-TDP and ALS pathology that also demonstrate differentiation and overlap in TDP-43 between these two phenotypes (i.e. the regional burden of TDP-43 in the anterior cingulate cortex has been shown to differentiate between ALS and bvFTD but >70% of FTLD cases without ALS demonstrate TDP-43 in the motor cortex) and that are rarely/not found to demonstrate TDP-43 in cognitively normal individuals and AD cases [6, 8, 9, 18, 30, 35].

### Quantitation of TDP-43 pathologies

**Fig. 1:**
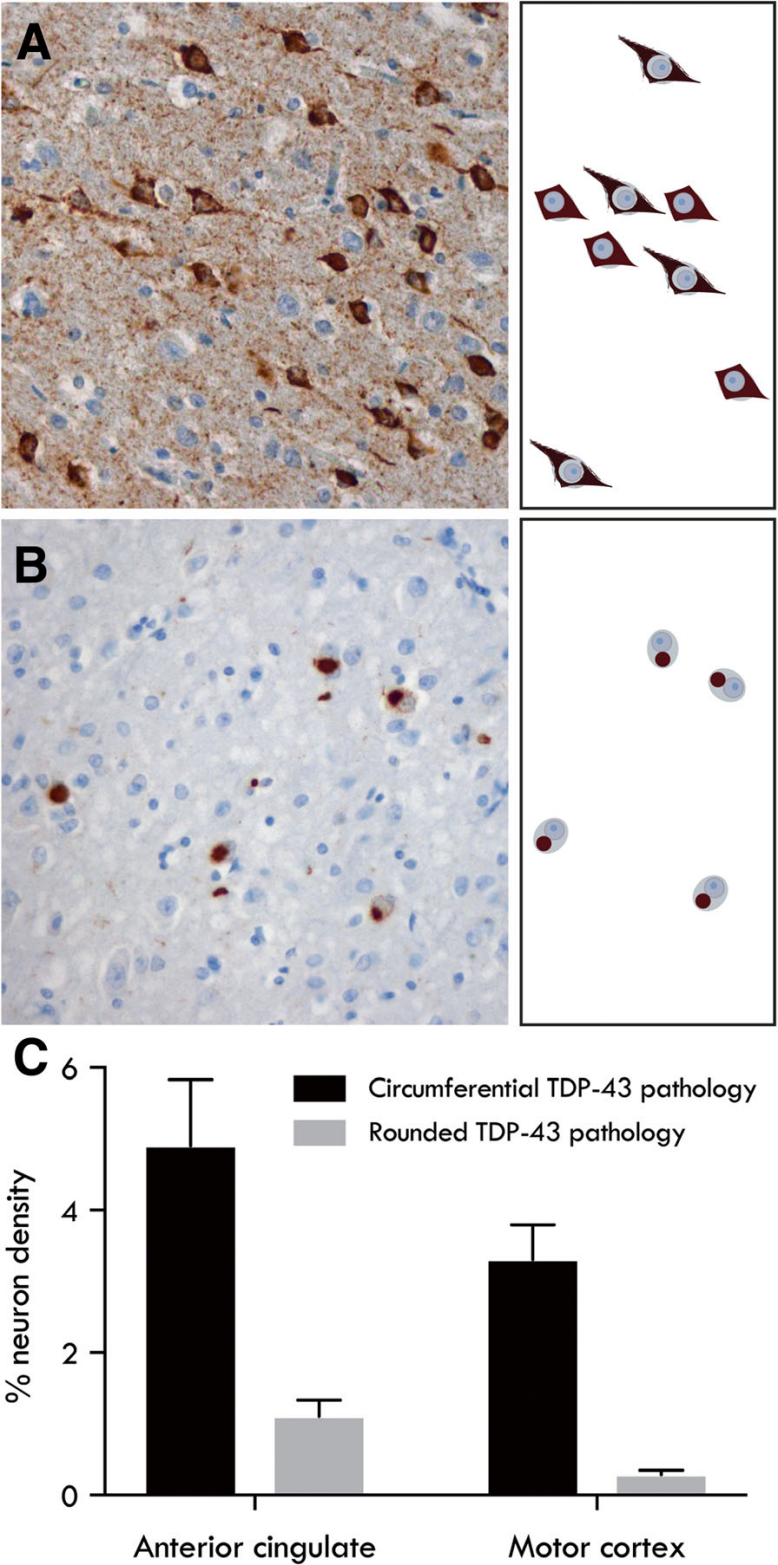
Regional TDP-43 morphologies: Micrograph and schematic of the characteristic (**a**) circumferential TDP-43 pathology and (**b**) rounded TDP-43 pathology. **c** Mean (± SE) circumferential and rounded TDP-43 inclusions identified in the anterior cingulate cortex and motor cortex across all cases

Formalin-fixed, paraffin-embedded tissue blocks for each region-of-interest were sectioned at 10μm and immunostained with the anti-phospho TDP-43 monoclonal antibody (1:80,000, TIP, PTD-MO1, Cosmo Bio). All slides were counterstained with haematoxylin for quantitation of cortical neuronal populations, as described previously [31]. Briefly, two strips of cortex, 500μm wide through the entire cortical thickness from the pial surface to white matter were sampled in each cortical section and cortical neurons with and without TDP-43 aggregates were counted at ×200 magnification using a 10 × 10 eyepiece graticule (500μm × 500μm) with standard inclusion (lower and left) and exclusion (upper and right) borders in contiguous, non-overlapping field. The density of neurons within each region was calculated for each case and the proportion of each TDP-43 morphology (circumferential, rounded, Fig. 1a, b) expressed as a percentage of these. Quantitation was performed by two raters blind to case details with an inter- and intra-rater variance of <5%.

### Statistics

Statistical analyses were performed using SPSS (IBM SPSS statistics version 24), with *p*-value <0.05 taken as significant. Specific analyses were conducted to determine:

1. The region with the greatest burden of TDP-43 morphologies using a univariate analysis with inclusion morphology and region as factors covarying for age and disease duration;
2. Any differences between cohorts (clinicopathological and genetic) in the regional density of different TDP-43 inclusions using ANOVA and posthoc tests for each region.
3. Any relationships between all variables using principal component factor analysis, including regional density of different TDP-43 inclusions (4 variables), neuronal density, cohort group, genetic mutation and demographic variables (age and disease duration).

## Results

### Regional TDP-43 morphology

TDP-43 pathology was identified in the anterior cingulate cortex of 87% ALS (*n* = 13) and all other cases, and in the motor cortex of 93% ALS (*n* = 14), 86% FTLD (*n* = 24) and 100% FTLD-ALS cases (*n* = 18). Two prevalent morphologies were identified – rounded TDP-43 neuronal inclusions and circumferential TDP-43 neuronal inclusions (Fig. 1). Assessment of the regional burden of TDP-43 morphologies revealed a significantly greater proportion of circumferential TDP-43 pathology compared to rounded TDP-43 pathology (F(1, 232) = 40.51, *p* < 0.001), with significantly more TDP-43 pathology identified in the anterior cingulate cortex compared to the motor cortex (F(1, 232) = 5.03, *p* < 0.05) across all cases. The same distribution of inclusions was observed in both regions with no interaction between morphology and regional burden of TDP-43 pathology.

### Clinicopathological and genetic comparisons

**Fig. 2:**
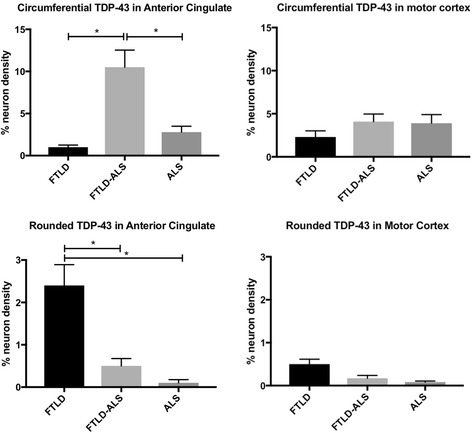
Mean (± SE) TDP-43 morphologies in the anterior cingulate cortex and motor cortex in frontotemporal lobar degeneration (FTLD), frontotemporal lobar degeneration and amyotrophic lateral sclerosis (FTLD-ALS) and amyotrophic lateral sclerosis (ALS). Significant differences were observed in the anterior cingulate cortex across clinicopathological cohorts, with FTLD-ALS cases demonstrating significantly more circumferential TDP-43 inclusions and FTLD cases demonstrating a significantly greater burden of rounded TDP-43 inclusions compared to other groups. *p < 0.05

Previous studies [30] have shown that the burden of TDP-43 pathology in the anterior cingulate cortex but not motor cortex differentiates bvFTD from ALS cases. Analysis of the morphology of the TDP-43 inclusions in the anterior cingulate cortex from the present series demonstrated significant differences across clinicopathological groups (F(2, 52) > 4.3, *p* < 0.05) with more circumferential TDP-43 inclusions in FTLD-ALS cases compared to ALS and FTLD cases (posthoc *p* < 0.001) and significantly more rounded TDP-43 inclusions in FTLD cases compared to ALS and FTLD-ALS cases (posthoc *p* < 0.005) (Fig. 2). The presence or type of genetic mutation did not influence this result (group (F(2, 52) > 0.59, *p* > 0.2), interaction (F(1, 52) > 0.2, *p* > 0.3)). In contrast, no significant cohort differences were observed in the densities of the different TDP-43 morphologies in the motor cortex (*p* > 0.5 for all clinicopathological and genetic groups (F(2,54) > 0.7, *p* > 0.2).

Principal component factor analysis was used to assess all relationships between variables across all cases and loading scores >0.6 was considered significant. Three separate factors accounted for >65% of variance. There were significant relationships between clinicopathological group (0.84 loading), the presence and type of genetic mutation (−0.71 loading), disease duration (0.61 loading) and % rounded TDP-43 inclusions (>0.63 loading) (factor 1, 34% of the variance). This supports the construct that rounded inclusions differentiated the FTLD group (Fig. 2) which had the majority of genetic cases, and that these inclusions increased over time. Factor 2 accounted for 18% of the variance and showed that age (−0.74 loading) was associated with the % circumferential TDP-43 inclusions in the motor cortex (0.71 loading) consistent with the older age of many of the ALS cases (Table 1). The % circumferential TDP-43 inclusions in the anterior cingulate cortex accounted for 15% of the variance but was not significantly related to any other variable as the density of these inclusions was increased only in a single clinicopathological group (FTLD-ALS, Fig. 2). No relationships between neuron density and % TDP-43 morphologies was observed.

## Discussion

The present study identified two prevalent TDP-43 inclusion morphologies in the anterior cingulate cortex and motor cortex of FTLD, FTLD-ALS and ALS cases – circumferential TDP-43 neuronal inclusions and rounded TDP-43 neuronal inclusions (Fig. 1). Quantitative analyses revealed significant differences in the burden of these TDP-43 inclusion morphologies in the anterior cingulate cortex, but not in the motor cortex of these three clinicopathological cohorts. Specifically, FTLD-ALS cases were found to have a significantly greater burden of circumferential TDP-43 inclusions compared to FTLD and ALS cases, whereas FTLD cases revealed significantly more rounded TDP-43 inclusions compared to FTLD-ALS and ALS cases. The presence or type of genetic mutation did not influence results. These findings of distinct TDP-43 inclusion morphologies in the anterior cingulate cortex of FTLD and FTLD-ALS cases converges with a growing body of evidence suggesting the involvement of divergent pathomechanisms in these two clinical syndromes believed to sit on the same disease continuum.

The present quantitative analysis of TDP-43 inclusion morphologies in predilection cortical regions implicated in FTLD, FTLD-ALS and ALS cases demonstrates a significant amount of circumferential TDP-43 inclusions in the anterior cingulate cortex of FTLD-ALS cases only. Importantly, these circumferential TDP-43 inclusions demonstrate a striking resemblance to: 1) the TDP-43 granular neuronal cytoplasmic inclusions recently described in the frontal cortices of FTLD-TDP type B cases, all with co-existing ALS (Fig. 3e–h in [22]); 2) the TDP-43 neuronal cytoplasmic inclusions reported in the temporal cortex of ALS cases with cognitive impairment (Fig. 1 in [29]) and; 3) the granulofilamentous TDP-43 neuronal inclusions recently reported in the cortices of 7 FTLD cases [20]. In all 7 FTLD cases described by Lee et al., TDP-43 neuronal inclusions were also observed in the lower motor neurons, but this was not accompanied by obvious neurodegeneration in the spinal cord [20]. Importantly however, neuronal loss in the spinal cord of ALS cases has been shown to be apparent only in cases with moderate to severe TDP-43 inclusions [7]. Given that all 7 FTLD cases described by Lee et al. had very short survivals of 3 years from disease onset, it is possible that end-stage ALS may have been present in these cases, consistent with similar cases described previously [15]. The present findings converge with these recent pathological reports to provide compelling evidence that cortical TDP-43 inclusions refered to as cortical circumferential TDP-43 inclusions in this study, are a distinctive feature of FTLD-ALS. Importantly, these granulofilamentous circumferential TDP-43 inclusions described by Mackenzie et al. and Lee et al. were found to be hyperphosphorylated but not ubiquinated [20, 22]. A critical basis to the continuum theory is that FTLD and ALS share similar mechanisms linked to the abnormal hyperphosphorylation, ubiquitination and cleavage of pathological TDP-43. As such the association of non-ubiquitinatated circumferential TDP-43 inclusions with FTLD-ALS suggests the involvement of a pathomechanism that is distinct from FTLD cases, which demonstrate predominantly rounded ubiquitinated TDP-43 inclusions. This notion converges with a growing body of pathological and molecular evidence contesting the continuum hypothesis [5, 14, 25, 32].

The present findings of significant amounts of rounded but not circumferential TDP-43 inclusions in the anterior cingulate cortex of FTLD cases, and significant amounts of circumferential but not rounded TDP-43 inclusions in the anterior cingulate cortex of FTLD-ALS cases also suggest that difficulties in reliably distinguishing between FTLD-TDP type A and B, even when attempted by experienced neuropathologists [3], may be due in part to differences in the presence of co-existing ALS in FTLD cohorts examined by different groups. Although one group reported that all FTLD-ALS cases had the FTLD-TDP type B subtype [22], other groups have identified either subtypes A or B in their FTLD-ALS cohorts [8, 27]. Although previous immunoblot analyses have reported slight differences in the molecular species of sarkosyl-insoluble phosphorylated TDP-43 in FTLD-TDP type A and B cases, all of the FTLD-TDP type B cases included in that comparison had co-existing ALS, raising the question as to whether the removal of FTLD-ALS cases from that experiment and the current FTLD classification scheme would augment the broad pathological and molecular overlap currently already seen between FTLD-TDP type A and B cases [5, 32]. Principal component analyses in the present cohort revealed a significant relationship between rounded TDP-43 inclusions and clinicopathological group only, which is consistent with the finding of rounded TDP-43 inclusions in the FTLD cohort, and the circumferential TDP-43 inclusions in the overlapping clinicopathological FTLD-ALS cohort. The relationship between rounded TDP-43 inclusions, survival and genetic mutation is also in line with the significantly longer survival and higher incidence of genetic mutations in the FTLD cohort. Although it may be tempting to speculate that circumferential TDP-43 inclusions are an early phase of rounded TDP-43 inclusions, the identification of significant amounts of this distinct inclusion morphology at the end of the FTLD-ALS disease course suggests otherwise. Future studies comparing the morphology of TDP-43 inclusions in larger cohorts of clinically and genetically well-characterised FTLD cases without ALS are needed in order to compare and refine the current TDP-43 classification scheme, and determine whether all FTLD cases without ALS or type C morphology can be characterised into one homogenous FTLD-TDP subtype characterised by rounded TDP-43 inclusions.

Further to previous studies demonstrating no significant difference in the presence and severity of TDP-43 pathology in the motor cortices of FTLD, FTLD-ALS and ALS cases [8, 25, 30], the present study identified no significant differences in TDP-43 inclusion morphologies in this region in these three clinicopathological groups. It is important to note however, that given the relatively mild TDP-43 pathology identified in this region in the present study, subtle differences in TDP-43 morphologies in this region may not have been detected here. Future studies in other FTLD and ALS cohorts with more severe TDP-43 pathology in motor cortex as previously shown [8, 25] will be able to confirm if significant morphological differences in this predilection site are present. Also, consistent with a semi-quantitative analysis recently performed in pathological FTLD subtypes [22], a methodological issue warranting consideration in the current context is the relatively small numbers of sporadic FTLD cases. Although the present findings appear consistent with previous clinicopathological results [17, 22] and genetic mutations were not found to have any bearing on the present findings, replication of the present study in a larger sporadic cohort is needed in order to confirm the association between rounded TDP-43 inclusions in the anterior cingulate cortex and FTLD cases without ALS.

## Conclusions

In summary, the present non-biased quantitative analysis in a large series of TDP-43 proteinopathy cases has shown distinct TDP-43 inclusion morphologies in the anterior cingulate cortex of FTLD and FTLD-ALS cases. Circumferential TDP-43 neuronal inclusions were predominantly identified in the anterior cingulate cortex of FTLD-ALS cases, and rounded TDP-43 neuronal inclusions were primarly observed in the anterior cingulate cortex of FTLD cases. The present results converge with recent findings demonstrating heterogeneity in the ubiquitination of pathological TDP-43 protein in FTLD cases associated with ALS [20, 22] to suggest the involvement of a divergent pathmechanism in FTLD cases with ALS compared to those without ALS.
